# Ultrathin Planar Metasurface-based Acoustic Energy Harvester with Deep Subwavelength Thickness and Mechanical Rigidity

**DOI:** 10.1038/s41598-019-47649-9

**Published:** 2019-08-01

**Authors:** Meng Jin, Bin Liang, Jing Yang, Jun Yang, Jian-chun Cheng

**Affiliations:** 1 Collaborative Innovation Center of Advanced Microstructures and Key Laboratory of Modern Acoustics, MOE, Institute of Acoustics, Department of Physics, Nanjing University, Nanjing, 210093 P.R. China; 2 Key Laboratory of Noise and Vibration Research, Institute of Acoustics, Chinese Academy of Sciences, Beijing, 100190 P.R. China

**Keywords:** Acoustics, Materials for devices

## Abstract

Despite the growing attentions dedicated to the harvesting of acoustic energy that is a clean and renewable yet usually wasted energy source, the long wavelength of airborne sound still poses fundamental limits on the miniaturization of harvester devices and hinders practical applications. Here we present an ultrathin and planar acoustic energy harvester with rigidity. We propose a distinctive metasurface-based mechanism that reduces the effective wavelength to produce extraordinarily strong local energy within deep-subwavelength dimension and enable high-efficiently harvesting energy of incident airborne sound with considerably long wavelength. Our design idea is implemented by a foldy-structured metasurface capable of confining low-frequency energy within narrow channel at resonance, with a piezoelectric plate judiciously placed to converse acoustic to electric energy. The resulting device is downscaled to as thin as *λ*/63 while keeping flat shape and mechanical rigidity. We analytically derive the effective acoustical parameter of the unit cell, and verify the theoretical predictions via numerical simulations which shows the generation of the maximum output power at the prescribed working frequency. Our design with compactness and rigidity makes an important step towards the miniaturization and integration of acoustic energy harvesters and may have far-reaching implication in diverse applications ranging from microelectronic device design to wireless and self-powered active sensing.

## Introduction

The past few decades have witnessed considerable efforts dedicated to the study of energy harvesting that gather other forms of energy from ambient environment and convert it into electric energy^1–7^. Among various kinds of harvestable energies such as wind^1^, vibrations^2–4^ and so forth, acoustic energy^2,5–7^, as a clean, sustainable, ubiquitous yet commonly wasted energy source, has recently attracted increasing attention, with great application potentials in diverse scenarios, such as controlling noise and producing power for micro-devices. However, acoustic waves innately have long wavelength and low power density, posing a fundamental challenge on the downscaling of acoustic energy harvesting devices, which is crucial for the development of microelectronic devices and wireless sensors. The traditional designs of acoustic energy harvesters are usually based on Helmholtz resonators (HRs)^8–10^ and quarter-wavelength resonators^11,12^ for amplifying the otherwise low acoustic energy density to be converted into electric energy by using piezoelectric^2,10^ or electromagnetic^3,13^ methods. Although it is possible to improve the conversion efficiency such as by changing the shape of cavity^14,15^, the requisite large cavities or long tubes result in bulky devices with limited energy density, significantly hampering the practical applications. The attempts to go beyond the limit of natural materials by using artificial structures began with some designs incorporated with phononic crystals^16^. Later, the emergence of acoustic metamaterials with unconventional acoustical properties enables many extraordinary ways of sound wave manipulation^17–25^ and offers new possibilities for enhancing the capability of acoustic energy harvesters with reduced sizes^26–36^. Effective acoustic energy harvesting can be realized by using planar acoustic metamaterials containing defects^34^ or sandwiched between coiled metastructures^35^ but with relatively bulky dimensions. The introduction of hybrid resonance into membrane-type acoustic metamaterials allows sound absorption and energy conversion with devices that have deep-subwavelength thickness but are mechanically fragile^36^. Yet the usually low frequency of airborne acoustic wave (esp. audible sound stemming from the machine vibration that contains high mechanical energy but has extremely long wavelength) in diverse applications and high complexity of environments call for further miniaturization of acoustic energy harvesting devices with no sacrifice of the mechanical robustness. This makes it pivotal to explore the mechanism for realizing a high-efficiency and ultrathin acoustic energy harvester with rigidity which, however, still remains elusive despite the practical significance.

In this article, we make an attempt to address these issues by designing an ultrathin acoustic energy harvester that has deep subwavelength thickness, planar profile and high rigidity and is capable of harvesting energy of airborne sound with high efficiency. We propose a distinctive mechanism that reduces the effective wavelength to produce extraordinarily strong local energy density within a physical dimension much smaller than the wavelength in air, enabling high-efficiency energy harvesting for the incident low-frequency sound. As a practical implementation of this mechanism, a metasurface unit cell is designed that is composed of a folded structure for effectively confining low-frequency energy within narrow channel at resonance, and a piezoelectric plate is placed at the location of maximal strain energy for achieving high conversion from acoustic to electric energy. The resulting device hence features small footprint (*λ*/63 in thickness and *λ*/4.4 in sidelength), high rigidity and high efficiency. The effective acoustical parameter of the metasuface unit cell is analytically derived for simply yet precisely designing the geometry of energy harvester with prescribed working frequency. We verify the theoretical predictions via numerical simulations, and demonstrate numerically the performance of the designed energy harvester that produces maximal output power by the designed device as illuminated by airborne sound at the desired frequency.

## Results

### The mechanism of designed acoustic energy harvester

**Figure 1:**
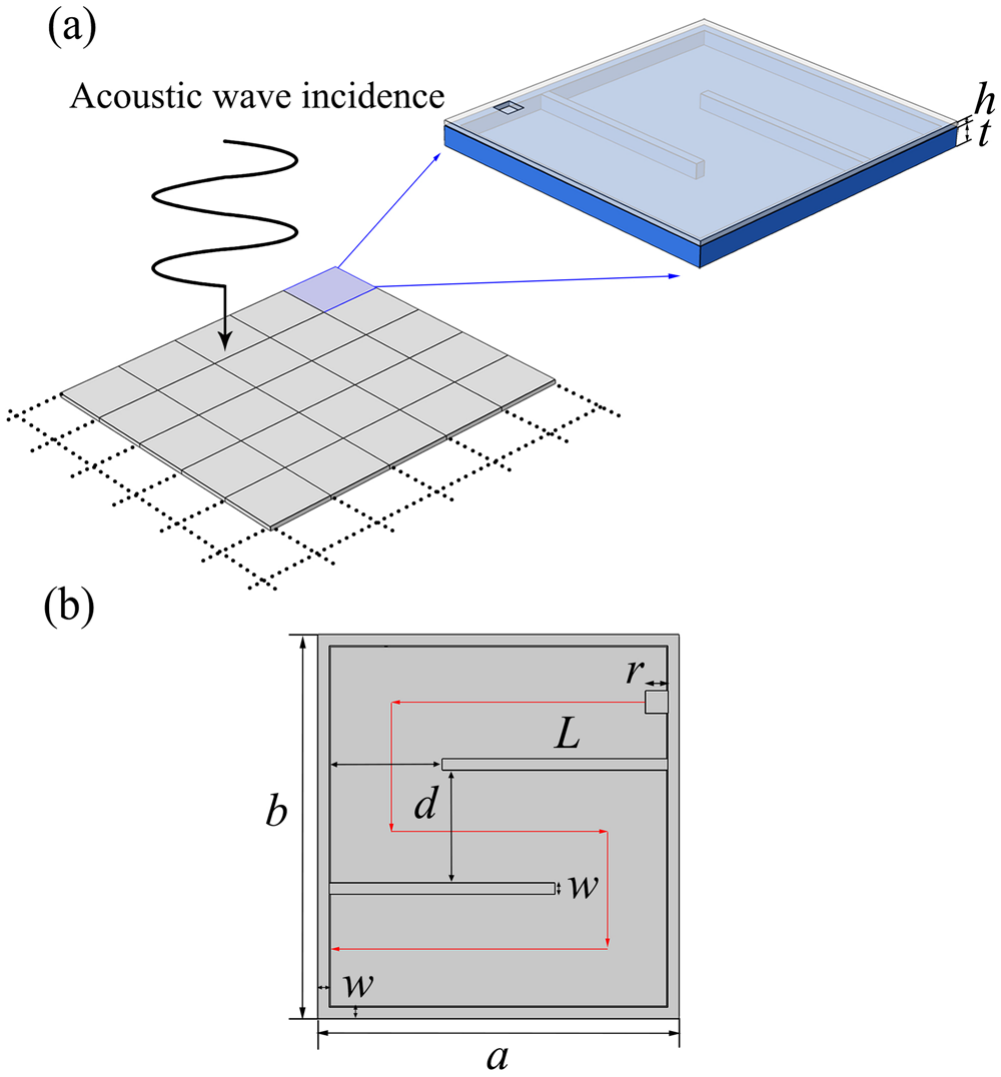
Schematic illustration and mechanism of our design. (**a**) Schematic illustration of the mechanism for designing ultra-thin planar acoustic energy harvester for absorbing and harvesting airborne sound with wavelength much longer than its physical dimension. Inset: Configuration of an individual unit cell of the designed metasurface, which is composed of a perforated plate and a labyrinthine-like channel. (**b**) Two-dimensional cross-section view of the metasurface unit cell which clearly illustrates the coiled channel and the substantially elongated effective propagation distance (marked by the red arrow) of the incident acoustic wave after entering the resonator.

Our design idea is schematically illustrated in Fig. 1(a). To break through the conventional limitations that the acoustic structures need to have comparable sizes to wavelength for effectively harvesting the incident acoustic energy, which usually rely on large cavities or long tubes that hinder the energy density enhancement and device miniaturization, we propose to downscale the harvesting device to deep-subwavelength scale by lowering the effective acoustic velocity inside. Due to the substantially shortened effective wavelength in comparison to the long wavelength in surrounding air, the device is capable to effectively interact with and produce strong localization of the incident acoustic energy despite its vanishing thickness, offering the possibility of energy harvesting in low frequency regime. For practically implementing this mechanism, we use an acoustic metasurface, a special class of metamaterial with a reduced dimension, to give rise to an abrupt reduction in the effective wavelength and, based on this, couple it with a piezoelectric (PZT) plate for converting acoustic energy into electricity high-efficiently. The designed metasurface is schematically shown in Fig. 1(a). The front cover of the metasurface is a plate (*a* in length, *b* in width, *h* in thickness) perforated with a squared opening (*r* in sidelength). Solid plates are inserted crossly to form air channel in the cavity (*t* in thickness). Due to the huge contrast in acoustical impedances between air and solids and the scalar wave nature of airborne sound, the incident wave will propagate along a zigzagged path (as marked by the red arrow shown in Fig. 1(b)) much longer than its physical dimension in the absence of cut-off frequency after entering the opening, leading to dramatic reduction in the effective sound velocity and subsequent amplification in the local energy density required for harvesting the acoustic energy.

### Analytical model of the acoustic metasurface

In order to maximize the energy conversion efficiency, however, we need to ensure that the incident acoustic energy effectively enters the proposed ultrathin acoustic energy harvester by judiciously designing the geometries. Hence we first give a theoretical analysis of the acoustical model of the metasurface and analytically derive its effective acoustical parameters, for obtaining insight to the physics underlying our mechanism as well as simplifying the design of the energy harvesting device.

The acoustical model of the metasurface unit cell is composed of a front perforated plate and a coiled channel attached to it. For simplification, the coiled air channel is acoustically treated as a straight long tube with effective length ${l}_{eff}$, which corresponds to the effective propagation distance in the labyrinthine-like channel. Then the effective surface acoustic impedance of the metasurface is calculated by

1 $${Z}_{s}={Z}_{o}+{Z}_{c}$$

where ${Z}_{0}$ and ${Z}_{c}$ represent the surface impedance of the opening and the coiled channel, respectively.

Considering air flow friction on the surface and sound radiation at both ends, the relative specific acoustic impedance of the perforated plate is^37^

2 $${Z}_{o}=\frac{8\eta h}{\sigma {\rho }_{0}{c}_{0}{{r}_{0}}^{2}}(\sqrt{1+\frac{{K}^{2}}{32}}+\frac{\sqrt{2}K{r}_{0}}{4h})+j\frac{\omega h}{\sigma {c}_{0}}(1+\frac{1}{\sqrt{9+{K}^{2}/2}}+1.7\frac{{r}_{0}}{h})$$

with

3 $$K=r\sqrt{\frac{\omega {\rho }_{0}}{\eta }}$$

where ${r}_{0}=\sqrt{{r}^{2}/\pi }$, ${\rm{\sigma }}={r}^{2}/{S}_{0}$ is ratio of perforation, $\eta$ is coefficient of viscosity. ${S}_{0}=ab$ and *h* represent the area and thickness of the perforated plate, respectively.

The normalized acoustic impedance of the coiled channel is given as

4 $${Z}_{c}=-j\frac{{S}_{0}}{{S}_{2}}\,\cot (\frac{\omega \cdot {l}_{eff}}{{c}_{0}})$$

where ${S}_{2}=d\cdot t$ is the cross section area of coiled channel.

Then the absorption coefficient of the designed acoustic metasurface can be calculated by

5 $${\rm{\alpha }}=\frac{4{x}_{s}}{{(1+{x}_{s})}^{2}+{y}_{s}^{2}}$$

where ${z}_{s}={x}_{s}+i{y}_{s}$, ${x}_{s}$ and ${y}_{s}$ represent the effective surface acoustic resistance and reactance, respectively. The resonance occurs when

6 $$\frac{\omega h}{\sigma {c}_{0}}(1+\frac{1}{\sqrt{9+{K}^{2}/2}}+1.7\frac{{r}_{0}}{h})=\frac{{S}_{0}}{{S}_{2}}\,\cot (\frac{\omega \cdot {l}_{eff}}{{c}_{0}})$$

Equations (5) and (6) provide simple relationship between the absorption coefficient of metasurface and its geometry, suggesting that the working frequency of the resulting device is dependent of multiple structural parameters. This helps to substantially simplify the parameter design for meeting the requirements on the working frequency and specific parameters such as device thickness and width. In the current design, the geometric parameters are preset as *r* = 0.4 cm, *d* = 2 cm, *L* = 4 cm, *w* = 0.2 cm, *t* = 0.32 cm, *h* = 0.1 cm respectively such that the device is targeted to harvest the energy of airborne sound with frequency of approximately 1303 Hz, which is within the frequency regime most sensitive to human ear, and the thickness is compressed to approximately $\lambda /63$.

### The performance of the ultrathin acoustic energy harvester

**Figure 2:**
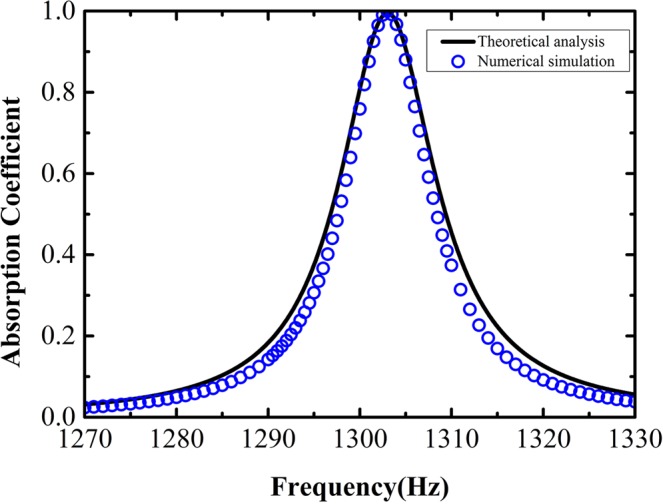
Results of the absorption coefficient. The black line is result obtained from theoretical analysis and blue circles are from numerical simulation.

Based on the above derivations, we can obtain the absorption coefficient of the designed acoustic metasurface itself and plot the result in Fig. 2. The numerical simulation result is also provided for comparison, which agrees quite well with the theoretical prediction. The result shows that the designed metasurface with the chosen geometrical parameters yields strongest interaction with the incident acoustic wave around 1303 Hz, where one can also expect the best performance of the whole acoustic energy harvester, as will be demonstrated later.

**Figure 3:**
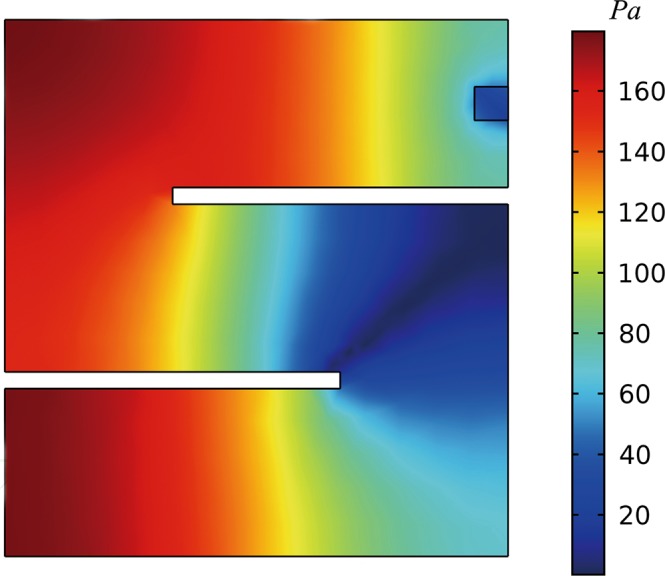
Result of acoustic pressure. Two dimensional view of the acoustic pressure distribution in the ultrathin acoustic metasurface at 1303 Hz.

**Figure 4:**
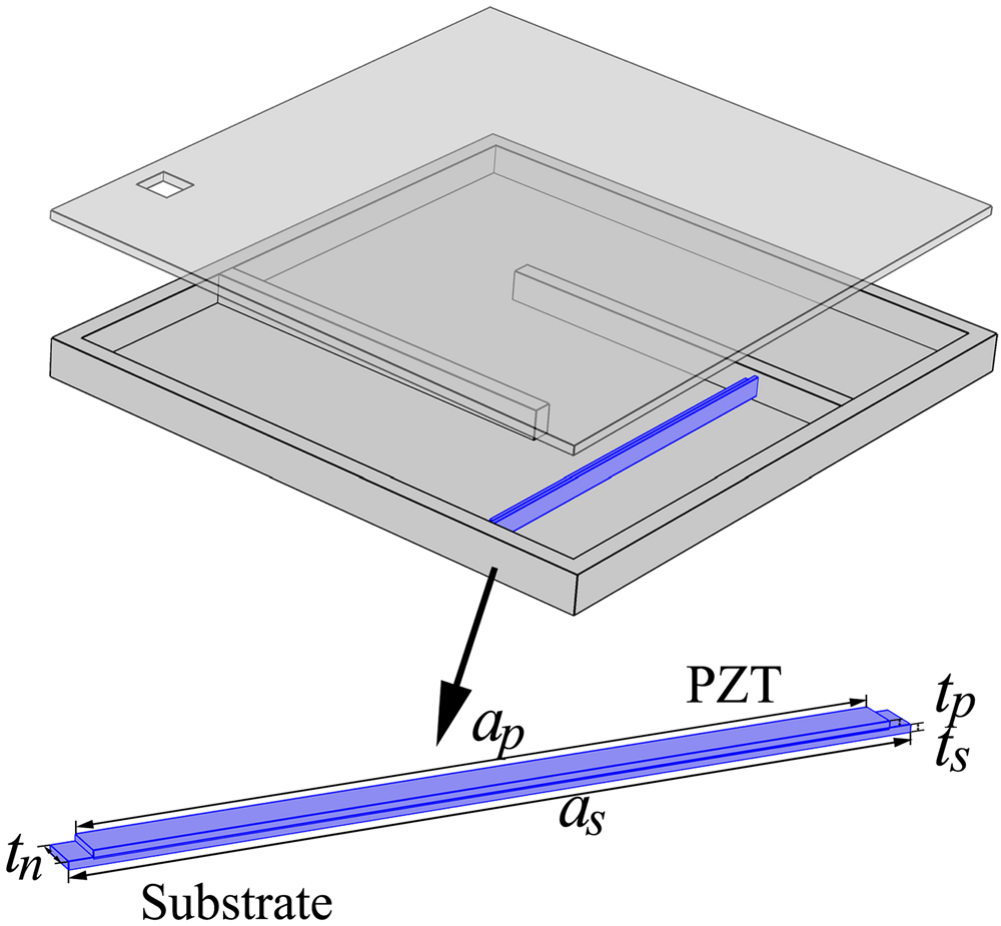
Sketch of the ultrathin acoustic energy harvester. The design is composed of a labyrinthine resonator and a piezoelectric plate with a PZT layer and a substrate patched under PZT.

To form the acoustic energy harvester, we need to incorporate the metasurface with energy conversion from acoustic energy to electricity. This can be realized by using a PZT plate which, however, must be appropriately placed for obtaining the optimal power output. From the numerical results of the spatial distribution of acoustic pressure inside the metasurface at 1303 Hz when incident acoustic pressure is 2 Pa (100 dB) as depicted in Fig. 3, we clearly observe the desired remarkable enhancement of acoustic energy density in the whole narrow channel and particularly strong localization around the first turning of coiled channel, almost two orders of magnitude higher than the energy density of the incident wave. Such a substantial amplification of local energy density stems from the strong low-frequency resonance that occurs in the designed coiled channel with an effective length much longer than its physical dimension in the thickness direction, as evidence by the spatial variation in the pressure amplitude caused by the inference between the incident and reflected waves in the channel shown in Fig. 3, and enables high-efficiency interaction between the incident acoustic energy and such deep-subwavelength device. Thus the PZT plate, which is chosen to have a narrow and thin geometry and tuned to resonate at 1303 Hz correspondingly, is located here to obtain maximum strain energy as excited by the incident acoustic wave. The schematic of the resulting ultrathin acoustic energy harvester is shown in Fig. 4. Notice that the whole structure is underpinned by the outer walls and multiple solid plates within the cavity, which can be fabricated with any solids due to the huge impedance contrast to the surrounding air, with no need to include fragile components such as resonating membranes. This endows our designed device with high mechanical robustness and ensures their application potentials in many important situations calling for mechanically rigid devices. Owing to the simple configuration and high design flexibility, our designed device composed of several subwavelength plates can be manufactured from any constituent solids with sufficiently high rigidity and can be fabricated precisely via 3D printing technique for experimental realization^26^.

**Figure 5:**
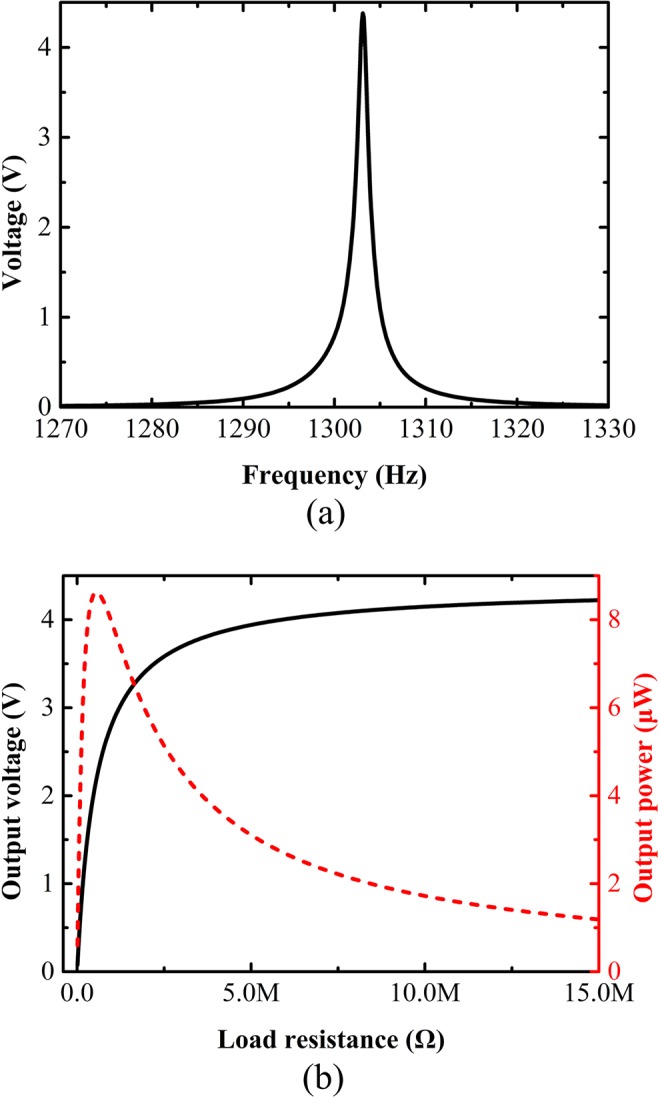
Output results of ultrathin acoustic energy harvester. (**a**) Simulated open circuit voltage of the piezoelectric plate as a function of frequency. (**b**) Simulated output voltage (black solid line) and electric power (red dashed line) of the piezoelectric plate versus the load resistance at 1303 Hz.

We demonstrate the effectiveness of our designed ultrathin acoustic energy harvester via numerical simulations. In the simulation, the length, width and thickness are tuned to be 38.57 mm, 2.04 mm and 0.26 mm respectively for the PZT layer, and 42 mm, 2.04 mm and 0.39 mm respectively for the substrate. Both ends of the substrate are fixed for vibration. The upper and below surface of PZT layer are set as floating potential and connecting to ground, respectively. Figure 5(a) shows the simulated frequency dependence of the open circuit voltage generated by the PZT plate and reveals that a peak voltage occurs at 1303 Hz as expected. At the peak, the thickness of the design is approximately ${\rm{\lambda }}/63$ while the sidelength of the design is ${\rm{\lambda }}/4.4$. We have also numerically examined the acoustic fields with and without the PZT plate and proved that its presence will not disturb appreciably the acoustic pressure distribution and resonance frequency.

For power conversion, a loading resistance needs to be applied to PZT layer. Figure 5(b) illustrates the simulated output voltage and electrical power at 1303 Hz as functions of the loading resistance connected to the upper surface of PZT layer, which is numerically calculated by coupling the circuit and electrostatic modules. It is seen that as the resistance increases, the output voltage increases rapidly to maximum value 4.4 V and becomes flat at last, while the output electrical power reaches a peak of 8.6 $\mu {\rm{W}}$ at loading resistance of 560 k${\rm{\Omega }}$ and then decreases, demonstrating the unique functionality of our designed device to produce high voltage and energy density despite deep-subwavelength dimension. We have also numerically verified that when the conversion efficiency reaches its maximum, the reflection coefficient of the metasurface is approximately 0.15, which indicates that our designed device has entered the device and been effectively harvested and the undesired scattering wave can be eliminated high-efficiently.

Our strategy also enables further downscaling of the device by redesigning the geometries for the metasurface and PZT plate simultaneously, at the cost of reducing working bandwidth. For potential application in practice, a hybrid structure containing multiple individually-designed unit cells can be introduced to enable richer functionality of the device, such as bandwidth expansion and harvesting energy for acoustic sources with other wavefronts^25^.

## Discussion

In summary, we propose to design an ultrathin and planar acoustic energy harvester and demonstrate a metasurface-based implementation with thickness of 1/63 sound wavelength. By coiling up space, the thickness of acoustic energy harvester is compressed to deep subwavelength scale. Acoustic energy is localized strongly in narrow channels and the piezoelectric plate achieve large strain energy confinement at the designed working frequency. With the effect of viscous and thermal loss being taken into account, we verify via numerical simulations that the maximum output voltage and power of 4.4 V and 8.6 $\mu {\rm{W}}$ are acquired with a driving amplitude of 2 Pa (100 dB) at a resonance frequency of 1303 Hz. In addition, our mechanism allows arbitrary choice of the constituent solid for composing the metasurface, which adapts to various environments while keeping high mechanical robustness, which is not achievable with the membrane-based energy harvesters that have deep-subwavelength thickness yet are relatively fragile mechanically. Compared to conventional acoustic energy harvesters that need to have bulky size, low conversion efficiency or fragile structure, the proposed device bears the advantages of ultrathin thickness, planar profile, simple configuration, high efficiency and rigidity, which are highly desirable in practical scenarios and may significantly improve the potentials of miniaturization and integration for energy harvesting devices. We anticipate our design with capability and flexibility to open up possibility for the design of novel functional devices and their applications in a plethora of situations such as microenergy production and noise mitigation.

## Methods

Throughout the paper, the numerical simulations are performed by COMSOL Multiphysics^TM^ Version 5.3. The background medium is air, for which the mass density and sound speed are 1.21 kg/m^3^ and 343 m/s, respectively. The solid material of the metasurface in simulation is reasonable assumed to be rigid as compared to air. The material for PZT layer is chosen as PZT-8 with the piezoelectric constant *d*_31_ = −97pC/N and damping ratio ${\rm{\varsigma }}=0{\rm{.001}}$. The material for substrate is chosen as structural steel. The mass densities of PZT-8 and substrate are 7600 kg/m^3^ and 7850 kg/m^3^, respectively. Plane wave radiation boundary condition is imposed on the incident boundaries to simulate spatial distribution of acoustic pressures. Thermoacoustic module is applied in the channel and squared opening to simulate viscous and thermal loss.
